# Hereditary Gastrointestinal Tumor Syndromes: When Risk Comes with Your Genes

**DOI:** 10.3390/cimb46070385

**Published:** 2024-06-26

**Authors:** María Jesús Fernández Aceñero, Cristina Díaz del Arco

**Affiliations:** 1 Department of Legal Medicine, Psychiatry and Pathology, School of Medicine, Complutense University of Madrid, 28040 Madrid, Spain; 2 Department of Pathology, Hospital Clínico San Carlos, Health Research Institute of the Hospital Clínico San Carlos (IdISSC), 28040 Madrid, Spain

**Keywords:** next-generation sequencing, familial syndromes, cancer, gastrointestinal tract, management, gastric cancer, colorectal cancer, genetic variant, mutation, hereditary

## Abstract

Despite recent campaigns for screening and the latest advances in cancer therapy and molecular biology, gastrointestinal (GI) neoplasms remain among the most frequent and lethal human tumors. Most GI neoplasms are sporadic, but there are some well-known familial syndromes associated with a significant risk of developing both benign and malignant GI tumors. Although some of these entities were described more than a century ago based on clinical grounds, the increasing molecular information obtained with high-throughput techniques has shed light on the pathogenesis of several of them. The vast amount of information gained from next-generation sequencing has led to the identification of some high-risk genetic variants, although others remain to be discovered. The opportunity for genetic assessment and counseling in these families has dramatically changed the management of these syndromes, though it has also resulted in significant psychological distress for the affected patients, especially those with indeterminate variants. Herein, we aim to summarize the most relevant hereditary cancer syndromes involving the stomach and colon, with an emphasis on new molecular findings, novel entities, and recent changes in the management of these patients.

## 1. Introduction

Despite recent campaigns for screening and the latest advances in therapy and molecular biology, gastrointestinal (GI) neoplasms remain among the most frequent and lethal human tumors [1]. Most GI neoplasms are sporadic, but there are some well-known familial syndromes associated with a predisposition to the development of GI cancer. The increasing use of next-generation sequencing (NGS) and revolutionary genetic technologies has led to the discovery of many new pathogenic or likely pathogenic variants of genes associated with GI and other human cancers [2]. The aim of this report is to review the genetic basis of both well-known syndromes and some of the most novel ones associated with gastric cancer (GC) and colorectal cancer (CRC), highlighting how this knowledge influences disease prevention and management.

## 2. Gastric Cancer

GC is the fifth most common cancer and the fourth leading cause of cancer-related deaths worldwide [3]. Its incidence varies geographically, being higher in Asian countries and lower in Western countries, where it is often diagnosed at advanced stages and has a five-year survival rate under 30% [4,5]. GC is a heterogeneous disease that can be classified according to various criteria. Microscopically, most GCs are adenocarcinomas. The most commonly used histological classifications are those published by Laurén and the World Health Organization [6]. Laurén’s classification identifies intestinal and diffuse types of GC. This classification will be used throughout this article due to its prevalence in the literature and its association with clinicopathological and prognostic factors. Laurén’s intestinal type forms tubular or glandular structures, whereas the diffuse type has poorly cohesive cells forming small nests or rows with little or no gland formation. In some cases, these cells exhibit a signet-ring morphology, with abundant cytoplasmic mucin and eccentrically displaced nuclei [7].

### Familial and Hereditary Gastric Cancer

Most GC cases are sporadic, associated with somatic molecular alterations, while a small subset shows familial aggregation and is linked to hereditary syndromes caused by germline mutations. The familial aggregation of GC is estimated at 5–10%, with hereditary GC accounting for 1–3% of cases [8,9].

**Figure 1:**
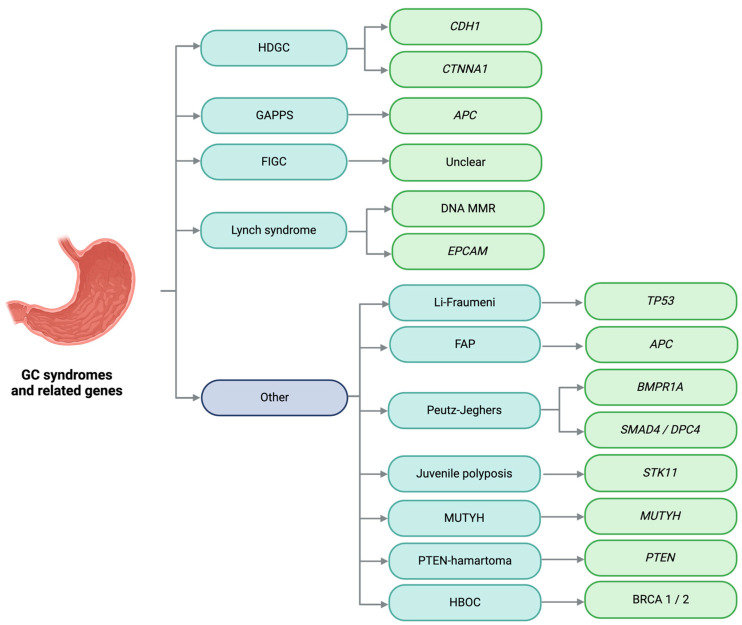
Hereditary gastric cancer syndromes and related genes. GC: gastric cancer; HDGC: hereditary diffuse gastric cancer; GAPPS: gastric adenocarcinoma and proximal polyposis of the stomach; FIGC: familial intestinal gastric cancer; FAP: familial adenomatous polyposis; MUTYH: *MUTYH*-associated polyposis, PTEN-hamartoma: *PTEN* hamartoma tumor syndrome; HBOC: hereditary breast and ovarian cancer; DNA MMR: DNA mismatch repair genes. Image created with Biorender.com (https://www.biorender.com/, accessed on 1 May 2024) under an individual license (Dr. Díaz del Arco).

**Figure 2:**
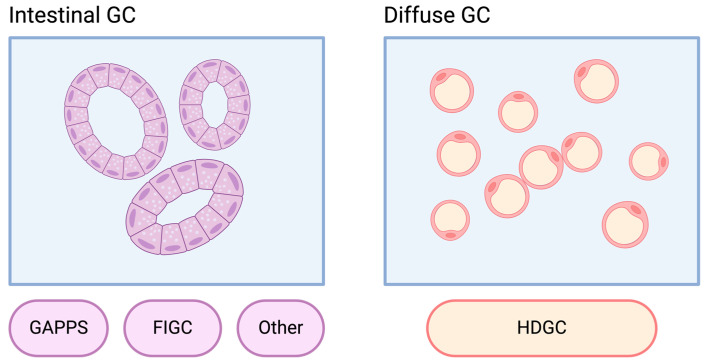
Laurén subtypes predominantly linked to hereditary gastric cancer syndromes. GC: gastric cancer; GAPPS: gastric adenocarcinoma and proximal polyposis of the stomach; FIGC: familial intestinal gastric cancer, HDGC: hereditary diffuse gastric cancer. Image created with Biorender.com (https://www.biorender.com/, accessed on 1 May 2024) under an individual license (Dr. Díaz del Arco).

Notable syndromes predominantly associated with GC include hereditary diffuse GC, gastric adenocarcinoma and proximal polyposis of the stomach, and familial intestinal GC [10,11]. Additionally, GC can occur in other syndromes such as Li–Fraumeni, familial adenomatous polyposis, Lynch syndrome, Peutz–Jeghers syndrome, juvenile polyposis syndrome, *MUTYH*-associated polyposis, hereditary breast and ovarian cancer, or *PTEN* hamartoma tumor syndrome [9]. Figure 1 presents the main genes associated with GC hereditary syndromes. The majority of these syndromes are most commonly associated with intestinal GC, except for hereditary diffuse GC (Figure 2). However, diffuse GC may be found in small proportions across most hereditary syndromes. Lynch syndrome, familial adenomatous polyposis, Peutz–Jeghers syndrome, juvenile polyposis syndrome, *MUTYH*-associated polyposis, and *PTEN* hamartoma tumor syndrome will be discussed in the colorectal section (Section 12 and Section 21).

## 3. Hereditary Diffuse Gastric Cancer

Hereditary diffuse GC is an autosomal dominant syndrome characterized by the early onset of diffuse GC and invasive lobular breast cancer [12]. Familial aggregation of diffuse GC was first noted in 1964 [13], and in 1998, the genetic basis of hereditary diffuse GC was discovered in Māori families from New Zealand [14]. Over time, similar findings have been observed in families worldwide from various ethnic backgrounds [15,16].

Currently, hereditary diffuse GC is defined by the presence of a pathogenic germline mutation in either *CDH1* or *CTNNA1* in an individual with diffuse GC or in a family with one or more cases of diffuse GC among first- or second-degree relatives [12]. *CDH1* mutations also predispose individuals to lobular breast cancer and non-syndromic cleft lip and palate [17]. Families that meet the criteria for hereditary diffuse GC genetic testing but do not have pathogenic variants in *CDH1* or *CTNNA1* are classified as hereditary diffuse GC-like, as seen in other syndromes [9].

### 3.1. Clinical Presentation

Initially described in 1999, hereditary diffuse GC criteria included (1) two or more cases of diffuse GC in first- or second-degree relatives, with at least one diagnosed before age 50, or (2) three or more cases of diffuse GC in first- and second-degree relatives regardless of age [18]. The latest guidelines update from 2020 incorporates both familial and individual criteria. Familial criteria for genetic testing include (1) two or more cases of familial GC regardless of age, with at least one being diffuse; (2) one or more cases of diffuse GC at any age and one or more cases of lobular breast cancer before age 70; or (3) two or more cases of lobular breast cancer in relatives before age 50 [12]. GC and breast cancer cases must be histologically confirmed, and the family members must be first- or second-degree blood relatives [17]. Indications for genetic testing in an individual depend on the age at diagnosis of cancer, presence or absence of bilateral breast cancer, ethnicity, associated phenotypic features, and other factors [12]. Additionally, the 2020 update recommends testing for *CTNNA1* in addition to *CDH1*.

In index cases, GC is generally diagnosed at an advanced stage, potentially presenting as linitis plastica [19]. Prophylactic gastrectomies often reveal precursor lesions such as in situ signet ring cell carcinoma or pagetoid spread of signet ring cells [20]. Intramucosal diffuse GC foci are also commonly found. In 95% of prophylactic gastrectomies, intramucosal carcinoma foci have been described, but the detection rate for in situ and pagetoid lesions seems to be lower (30%), suggesting intramucosal carcinoma might develop without a preceding preinvasive phase [21]. These lesions are associated with decreased E-cadherin expression on immunohistochemistry (IHC), which can appear as complete loss, mild membrane staining, or aberrant cytoplasmic or granular staining [22]. However, this staining is not sufficiently sensitive or specific to be used for screening patients for *CDH1* testing [23].

### 3.2. Gastric Cancer Risk

#### 3.2.1. Individuals with Pathogenic *CDH1* Variants

Penetrance is incomplete but high, with a significant risk of GC, estimated between 37 and 70% for men and between 25 and 83% for women [24,25,26,27]. Women with *CDH1* mutations also face a 42–55% risk of lobular breast cancer, leading to a cumulative cancer risk of 90% [28,29].

The age of GC onset varies widely, from 14 to 85 years, even within the same family [12]. The median age varies by study, with the most common range being between 30 and 50 years [30,31]. Recent reports indicate most cases are diagnosed before age 40 [8].

#### 3.2.2. Individuals with Pathogenic *CTNNA1* Variants

The causal relationship between *CTNNA1* and hereditary diffuse GC has been more recently recognized, so evidence on the associated risks of GC and breast cancer is still emerging. A recent study indicated a cumulative GC risk of 49–57% by age 80, similar to *CDH1* mutations [32]. The risk of breast cancer in these patients remains under investigation [9].

### 3.3. Molecular Basis

#### 3.3.1. *CDH1*

**Figure 3:**
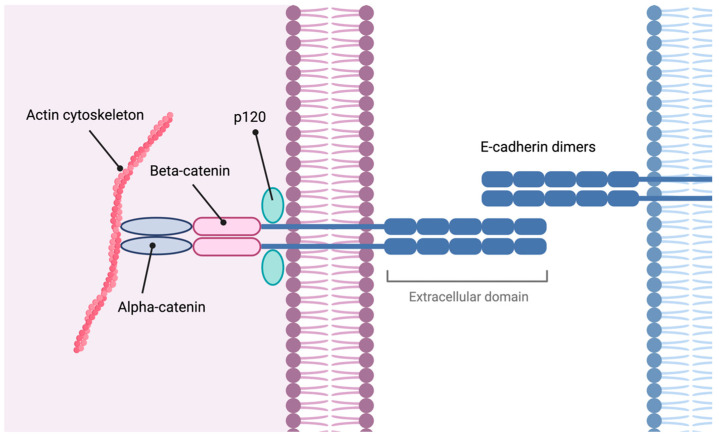
Adherens junction. E-cadherins have extracellular, transmembrane, and intracellular domains. They form homodimers, and the intracellular domain interacts with adaptor proteins such as p120 and catenins to connect with the actin cytoskeleton. Image created with Biorender.com (https://www.biorender.com/, accessed on 1 May 2024) under an individual license (Dr. Díaz del Arco).

The *CDH1* gene is a tumor suppressor that encodes E-cadherin, a transmembrane protein involved in adherens junctions of epithelial cells. It plays a critical role in cell–cell adhesion, tension sensing, and signal transduction [33]. Figure 3 illustrates the composition of these cell junctions.

In hereditary diffuse GC, alterations in the *CDH1* gene result in a loss of function [17]. *CDH1* variants are estimated to have a frequency of 5–10 per 100,000 births [12]. Mutations can be found throughout the coding sequence, with no identified hot spots [9,34,35]. According to the two-hit hypothesis, a somatic second-hit event causes biallelic inactivation, leading to the deregulation of E-cadherin and the development of diffuse GC or invasive lobular breast cancer [17].

E-cadherin plays a crucial role in maintaining cellular differentiation, polarity, and epithelial architecture [9]. Disruption of adherens junction components leads to loss of cell cohesion, promoting epithelial–mesenchymal transition (EMT), cell invasiveness, carcinogenesis, and development of metastasis [36].

**Figure 4:**
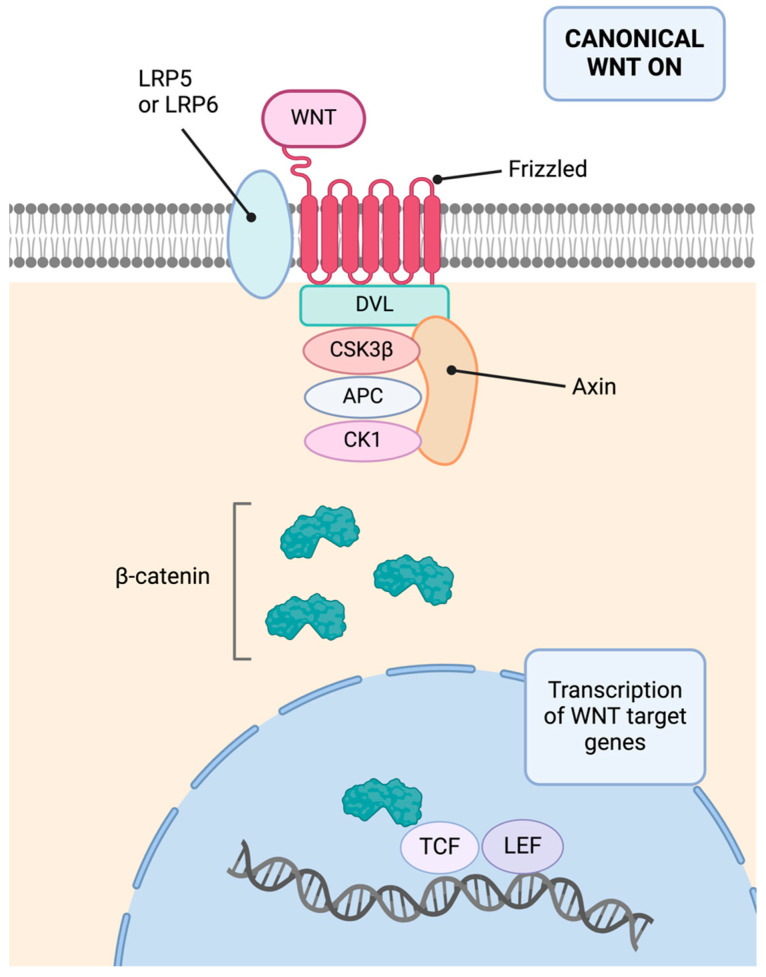
Activated canonical Wnt pathway. The Wnt protein binds to the extracellular domain of a Frizzled family receptor, alongside various co-receptors. Upon activation, it triggers a cytoplasmic cascade, leading to the accumulation of β-catenin, its translocation to the nucleus, and the transcription of target genes. Image created with Biorender.com (https://www.biorender.com/, accessed on 1 May 2024) under an individual license (Dr. Díaz del Arco).

In addition, E-cadherin sequesters β-catenin at the cell membrane, preventing its nuclear entry and cell proliferation promotion [37]. This inhibition of the Wnt/β-catenin pathway has a protective effect, as this pathway is also linked to carcinogenesis, cellular invasiveness, EMT, metastasis, and chemotherapy resistance [38,39]. Additionally, E-cadherin’s extracellular domain can interact with growth factors, inhibiting ligand-dependent activation of receptor tyrosine kinases and suppressing proliferative signals, as well as inhibiting the Hippo-YAP pathway [40]. Figure 4 presents a schematic of the activated canonical Wnt pathway.

#### 3.3.2. *CTNNA1*

*CTNNA1* encodes α-catenin, a protein also involved in adherens junctions. Mutations in this gene account for less than 2% of hereditary diffuse GC cases [41,42,43,44]. It is hypothesized that α-catenin alterations also occur after a second-hit event [43]. Previous studies have identified at least 25 mutations in *CTNNA1* in GC [42].

In respect of the structure and functions of *CTNNA1* and α-catenin, *CTNNA1* is located on chromosome 5q31 and is widely expressed in human tissues [45]. The α-catenin protein family includes three members: αE-catenin (epithelial), αN-catenin (neuronal), and αT-catenin (testis and heart) [9]. This family maintains the integrity of adherens junctions by anchoring the cadherin-β-catenin complex to the actin cytoskeleton, strengthening intercellular adhesion [46,47].

α-catenin has additional tumor suppressor functions beyond those related to intercellular junctions, as it inhibits critical pathways such as NF-κB, Hedgehog, and Wnt/β-catenin and regulates the Hippo-YAP pathway [48]. The NF-κB pathway is involved in cell proliferation, survival, differentiation, EMT, angiogenesis, metastasis, and immune response [49]. The Hedgehog pathway is associated with GI tumor development, maintaining tumor stem cells in a proliferative state or establishing a microenvironment essential for tumor growth [50]. Finally, the Hippo-YAP pathway has opposing roles in carcinogenesis, exhibiting both pro- and anti-oncogenic effects [51]. α-catenin sequesters YAP in the cytoplasm, inhibiting its function, and previous studies have shown that loss of α-catenin function can have pro-tumorigenic effects [48].

#### 3.3.3. Other Molecular Alterations

Previous studies have suggested that hereditary diffuse GC may be associated with alterations in other tumor suppressor genes [42,52]. For example, a relationship has been identified between alterations in genes involved in double-stranded DNA repair and diffuse GC (*PALB2*, *BRCA2*, possibly *RAD51C*, and *ATM*) [53]. However, most of these studies are at the research level, and the association of these mutations with GC risk and their pathogenicity needs to be demonstrated for clinical application [44]. Therefore, some authors emphasize the need for future studies to investigate the specific mechanisms of diverse genes implicated in diffuse GC [45].

### 3.4. Follow-Up and Treatment

For patients with *CDH1* alterations, endoscopic surveillance and prophylactic total gastrectomy are recommended. Gastrectomy is advised between the ages of 20 and 30, or 5 years before the youngest family member was diagnosed with GC, and is generally not recommended for those over 70 years old [12,54]. Post-surgery, in an ideal resource setting, examination of the entirety of the resected specimen is recommended. However, due to limitations in some laboratories, guidelines offer various options depending on the context [12].

When patients meet certain criteria, such as lacking a family history of GC, having variants of unknown pathogenicity, being under 20 years old, or declining or having contraindications for prophylactic gastrectomy, endoscopic surveillance may be performed [55]. Endoscopic examination should be conducted every 6–12 months, and any visible lesions should be biopsied along with random areas. A minimum of 30 biopsies from the antrum, transitional zone, body, fundus, and cardia is recommended, following the Cambridge protocol, a consensus approach [56]. To increase sensitivity, methods such as chromoendoscopy, endoscopic ultrasonography, or confocal endoscopic microscopy can be used [57,58]. The application of artificial intelligence techniques to endoscopic studies could enhance sensitivity, but more work is needed in this field to reach clinical application [59,60].

Some authors suggest that for asymptomatic carriers of *CTNNA1* mutations, annual endoscopic surveillance is recommended. However, due to the recently described risk of GC associated with pathogenic mutations of this gene, these alterations should probably be managed similarly to *CDH1* alterations [12].

## 4. Gastric Adenocarcinoma and Proximal Polyposis of the Stomach

Gastric adenocarcinoma and proximal polyposis of the stomach is a recently described autosomal dominant gastric polyposis syndrome [53]. It was first identified in 2012 in a large Australian family, along with two smaller families in the US and Canada [61]. The syndrome is characterized by the presence of over 100 polyps (though some family members have fewer) in the gastric body and fundus. These polyps are typically sessile and small (<1 cm), although they can grow larger [62,63]. The youngest patient diagnosed with this syndrome was 10 years old [61]. Gastric adenocarcinoma and proximal polyposis of the stomach exhibits phenotypic variability, with differences in age of onset, penetrance, and degree of dysplasia. Diagnosis is based on clinical and endoscopic criteria [64]. A colonoscopy is necessary to exclude attenuated familial adenomatous polyposis, and proton pump inhibitor use must be ruled out [65].

### 4.1. Gastric Cancer Risk

Gastric adenocarcinoma and proximal polyposis of the stomach carries a significant risk of GC, estimated between 12 and 25%, with incomplete penetrance [61,62,66,67]. It is commonly associated with intestinal-type GC, which tends to occur in areas with high-grade dysplastic fundic gland polyps and adenomatous polyps. Some authors suggest the risk may be lower due to the limited quality of the literature, as most cases are described in case reports rather than series [55]. The age of GC onset varies between 25 and 70 years [67,68].

### 4.2. Molecular Basis

The molecular alteration associated with gastric adenocarcinoma and proximal polyposis of the stomach was described in 2016, identifying a germline point mutation in the promoter 1B of the *APC* gene [66]. Subsequent research has detected various mutations in this promoter [69]. These mutations reduce the binding of the transcription factor Yin Yang 1, decreasing the transcriptional activity of the *APC* promoter [66]. In most cases, a second hit occurs via loss of the wild-type allele or truncating mutations [70]. Interestingly, similar mutations are found in rare families with familial adenomatous polyposis [66]. The functions of the *APC* gene are further discussed in the sections on familial adenomatous polyposis and *MUTYH*-associated polyposis (see below).

### 4.3. Follow-Up and Treatment

A personalized approach is recommended due to the interindividual heterogeneity in gastric adenocarcinoma and proximal polyposis of the stomach and the limited available data [70]. Recommendations are primarily based on expert opinion, and follow-up criteria vary by guidelines [64,71]. Some investigators suggest performing an upper endoscopy every 6–12 months, while others recommend follow-up based on the degree of polyposis: every 5 years if no polyps are present, every 3 years if fundic gland polyps without dysplasia are present, and individualized decisions for patients with over 100 fundic gland polyps [67,71]. Endoscopic screening should begin at age 15 or with the onset of dyspeptic symptoms, including a detailed examination of the polyps and surrounding mucosa with multiple biopsies of suspicious areas [71].

Diagnosing GC in the context of this syndrome is challenging because of the numerous polyps, some with adenomatous or dysplastic changes, which are indistinguishable endoscopically. In some cases, GC has only been diagnosed after prophylactic gastrectomy despite endoscopic surveillance [68]. Prophylactic gastrectomy is recommended at least for patients with dysplastic polyps [71]. However, given the limited correlation between endoscopic and histopathological findings, some authors advocate for performing gastrectomy on all gastric adenocarcinoma and proximal polyposis of the stomach patients without contraindications for surgery [72].

## 5. Familial Intestinal Gastric Cancer

Familial intestinal GC is an autosomal dominant syndrome associated with an increased risk of intestinal-type GC and other tumors, such as colorectal and breast cancer [11,73], in patients who do not present gastric polyposis or a genetic alteration associated with another syndrome [74]. It is important to note that data on this entity are very scarce, resulting in suboptimal quality of available evidence.

### 5.1. Clinical Presentation

The diagnostic criteria for familial intestinal GC were established in 1999 [18]. In countries with high incidence, familial intestinal GC is suspected when a family has three or more relatives with intestinal GC, with at least one being a first-degree relative, GC diagnosis in at least two generations, and at least one GC patient diagnosed before the age of 50. In countries with low incidence, the criteria include two scenarios: families with at least two first- or second-degree relatives with intestinal GC, one diagnosed before age 50, and families with three cases of intestinal GC regardless of the age of diagnosis [75]. Recent recommendations suggest relaxing these criteria, considering familial intestinal GC in any family with two cases of GC (one intestinal), regardless of the age of diagnosis [11].

### 5.2. Gastric Cancer Risk

The clinical spectrum of familial intestinal GC is not well defined and has not significantly changed in recent years [75]. A recent study suggested that GC appears later in familial intestinal GC than in hereditary diffuse GC and about 10 years earlier than sporadic intestinal GC [11]. The risk of GC in these families is not well established; however, a 2024 analysis in a Western country with a low incidence of GC observed a 4.9% incidence of GC and a 43.9% incidence of precursor lesions in patients undergoing endoscopic follow-up after familial intestinal GC diagnosis [74]. Another study found a 66% prevalence of GC in a cohort of 50 families meeting familial intestinal GC criteria [11].

### 5.3. Molecular Basis

The genetic cause of familial intestinal GC remains unclear [76,77]. In one family, heterozygous mutations in the *IL12RB1* gene were identified [76]. NGS studies have identified other potential genes such as *TP53*, *BRCA2*, *BRCA1*, *MSH2*, and *BRIP1* [75]. Microsatellite instability (MSI) has been observed in 38% of cases [11]. However, the relationship between these genes and the disease is not clear. Carvalho et al. suggested that familial intestinal GC might be a polygenic disease, as their 2021 study on this disease did not find alterations in high-penetrance genes [11].

### 5.4. Follow-Up and Treatment

Endoscopic surveillance is recommended; however, due to the limited information on this entity, standardized follow-up protocols do not exist and recommendations vary. Some authors suggest starting endoscopic surveillance at age 60, while others recommend starting at age 40, five years before the youngest GC diagnosis in the family, or ten years before said diagnosis [78,79]. The recommended interval between endoscopies also varies, ranging from annually to every three years or every five years [74].

As with all syndromes related to intestinal GC, preventive eradication of *Helicobacter pylori* is recommended. This recommendation is supported by results from Llach et al., who observed that *H. pylori* infection was the only factor independently associated with the development of precursor lesions in this syndrome [74].

## 6. Li–Fraumeni Syndrome

Li–Fraumeni syndrome is a rare autosomal dominant syndrome caused by germline mutations in the *TP53* gene [53]. It is characterized by familial aggregation of tumors in individuals under 45 years of age, predominantly including sarcomas, breast cancer, brain tumors, and adrenal cancer, which account for 80% of cases [80]. First described in 1969 within four families exhibiting early-onset tumors, Li–Fraumeni syndrome has since been identified in 24 families comprising 151 relatives with cancer [81,82]. In 1990, the molecular cause was discovered through the identification of germline missense mutations in *TP53* in five families, with typical findings [83].

### 6.1. Clinical Presentation and Gastric Cancer Risk

The French Li–Fraumeni syndrome working group has revised the criteria for Li–Fraumeni syndrome to encompass various clinical presentations linked to germline *TP53* mutations and to improve clinical identification. The new “Chompret criteria” outline three primary scenarios indicative of Li–Fraumeni syndrome: familial presentation, where a proband has an Li–Fraumeni syndrome-associated tumor under 46 years old and a first- or second-degree relative has an Li–Fraumeni syndrome-associated tumor under 56 years old or has multiple tumors; multiple primary tumors, with at least two belonging to the Li–Fraumeni syndrome spectrum, the first of which developed before 46 years old; and rare cancers such as adrenocortical carcinoma or choroid plexus carcinoma, regardless of family history [84]. The risk of GC in individuals with Li–Fraumeni syndrome is relatively low. The most extensive series identified this tumor in 5% of patients and 22.6% of families with Li–Fraumeni syndrome, with an average age of onset at 43 years [85]. According to the International Agency for Research on Cancer database, the individual and familial risk of GC in this syndrome is 3.3% and 5.9%, respectively [86]. Li–Fraumeni syndrome-associated GC can be either intestinal or diffuse, although intestinal GC appears to be more common [85].

### 6.2. Molecular Basis

**Figure 5:**
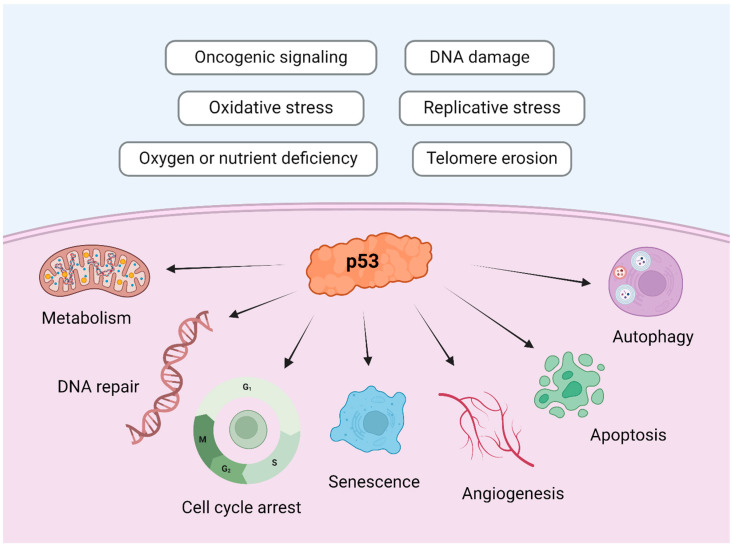
Physiological roles of p53. The p53 protein is a transcription factor that responds to various stimuli and, as a result, can induce cell cycle arrest, DNA repair, senescence, or programmed cell death, among other processes. Image created with Biorender.com (https://www.biorender.com/, accessed on 1 May 2024) under an individual license (Dr. Díaz del Arco).

Loss-of-function mutations in *TP53* lead to an accumulation of genetic alterations, resulting in tumor formation [87]. *TP53* is located on the short arm of chromosome 17, spanning 20 kb with an extensive intronic region [88]. It is a tumor suppressor gene, also known as “the guardian of the genome”, and it is involved in DNA repair, genomic stability, senescence, autophagy, cell-cycle control, cell differentiation, angiogenesis, and apoptosis [89]. Figure 5 illustrates the principal physiological functions of the p53 protein.

### 6.3. Follow-Up and Treatment

In this syndrome, surveillance with physical exams, lab tests, and frequent imaging using a combination of whole-body, brain and breast magnetic resonance imaging, mammography, abdominal and pelvic ultrasound, and colonoscopy is recommended, as it has been shown to improve patient survival [90].

On the other hand, the management of upper GI tumors in Li–Fraumeni syndrome is not standardized due to limited available data [91,92]. Generally, upper endoscopy is recommended from the age of 25 or 10 years before the first case of GC in the family, but the cost–benefit of endoscopic screening is debated [53,93]. Interestingly, Tjandra et al. reported an excess of gastro-esophageal junction tumors in *TP53* mutation carriers, emphasizing a detailed endoscopic examination of the esophagus and gastro-esophageal junction in these patients [93].

## 7. Hereditary Breast and Ovarian Cancer

Recent studies suggest that GC may be part of the spectrum of this syndrome, with both *BRCA1* and *BRCA2* mutations [94]. However, the risk of GC for each gene, as well as its pathogenesis and the need and protocol for GC screening are still under investigation [95].

## 8. Colon

CRC ranks as the second leading cause of cancer-related mortality worldwide. Approximately one-third of CRC cases exhibit familial clustering, but less than 20% are associated with germline mutations in cancer predisposition genes [96]. Non-syndromic CRC is defined as the clustering of CRC cases in a family without any apparent hereditary molecular abnormality. This clustering may be due to yet unidentified genetic alterations or to somatic tumors that aggregate in families because of a combination of environmental and common genetic factors [97].

In a recent review of nearly 12,000 patients with malignant tumors by Stadler et al., both tumor and germline mutation analyses were performed. Among these patients, nearly 2000 (10.5%) had CRC, with 72.4% presenting with metastatic disease. A germline mutation predisposing to cancer was found in 15.4% of the cases, many of which were potentially targetable [98].

Another study by You et al., focused on CRC patients under 50 years of age—a rapidly growing demographic—found that 67% of cases lacked a family history of cancer. Only 8% had deficiencies in mismatch repair (MMR) proteins, but a germline mutation analysis in 70% of the cohort (130 patients) revealed pathogenic variants in 19% of cases and variants of uncertain significance (VUS) in 18%. Notably, 12% of patients without a family history of disease and 13% of cases with intact MMR proteins carried pathogenic variants [99]. These findings suggest that germline mutation analysis can inform the management of both advanced-metastatic tumors and early-stage patients, aiding in risk reduction strategies.

**Figure 6:**
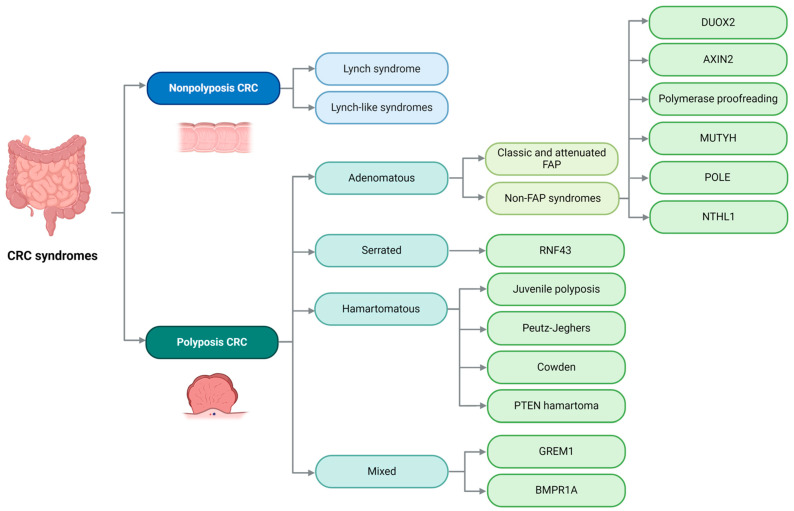
Main familial/hereditary syndromes of colorectal cancer. CRC: colorectal cancer; FAP: familial adenomatous polyposis. Image created with Biorender.com (https://www.biorender.com/, accessed on 1 May 2024) under an individual license (Dr. Díaz del Arco).

Historically, our understanding of the genetic basis of hereditary non-polyposis CRC, specifically Lynch syndrome, and hereditary polyposis CRC, including adenomatous, serrated, and hamartomatous polyposis, accounted for less than 7% of familial CRC clustering. However, NGS is expanding our knowledge of the mutations that can lead to CRC, adding complexity to this issue [100]. Figure 6 illustrates the various entities that will be discussed in this review.

## 9. Lynch Syndrome

Aldred Scott Warthin described the first case of what we now call Lynch syndrome in 1895 in a large family with a high incidence of carcinomas (the so-called “family G”) [101]. In 1971, Henry Lynch reported the results of genetic studies he performed on members of the original family G described by Warthin, confirming an autosomal pattern of inheritance with a high prevalence of GI and endometrial tumors, coining the term “Cancer Family Syndrome”. However, Lynch could not describe the genetic basis of this disease due to the technical limitations of the time. It was soon noted that some families had only CRC while others also had extraintestinal neoplasms, leading to the terms Lynch syndrome I and II, which later fell into disuse. In 1985, Lynch used the term hereditary non-polyposis CRC for this disease. In 2005, Douglas et al., working again with members of family G, found a specific mutation in the *MSH2* gene [102].

### 9.1. Clinical Presentation

Lynch syndrome occurs due to inherited genetic mutations that impair DNA MMR. It is associated with a wide variety of neoplasms, depending on the gene affected, which usually appear at a young age [103]. Lynch syndrome is associated with a high risk of CRC; endometrial cancer; and cancers of the ovary, stomach, small intestine, hepatobiliary tract, upper urinary tract, brain, and skin [104]. However, not all patients with mutations develop neoplasms. Both personal and familial history are important to define the risk of disease and the need for surveillance and screening [105]. In 1991, an international collaborative group established the Amsterdam criteria to suspect Lynch syndrome, emphasizing the importance of familial clustering of CRC [106]. According to these criteria, suspicion of Lynch syndrome arises when three relatives are affected by CRC, one of whom is a first-degree relative of the other two; CRC appears in at least two generations; and at least one member is diagnosed with CRC before the age of 50. Additionally, it is crucial that the family history shows no features of familial adenomatous polyposis. These criteria were revised in 1999 to include not only CRC but also other cancers associated with this disease. Later, the Bethesda criteria were proposed as an alternative for diagnosing Lynch syndrome [107].

### 9.2. Colorectal Cancer Risk

Although Lynch syndrome is the most frequent hereditary CRC syndrome, it represents less than 5% of all CRC cases. However, the risk of developing CRC for patients with Lynch syndrome is significantly increased compared to the general population (82% vs. 5%) [108]. The lifetime risk of developing CRC is highest for *MLH1* pathogenic variants (50% for men and 45% for women), followed by *MSH2* (45% for men and 40% for women), *PMS2* (20% for men and 15% for women), and *MSH6* (10% for men and 15% for women) [105].

### 9.3. Gastric Cancer Risk

The risk of GC in Lynch syndrome ranges from 6% to 13%, with variations depending on ethnicity and geographic location [109]. Approximately two-thirds of the reported cases correspond to intestinal GC.

### 9.4. Molecular Basis

Lynch syndrome is an autosomal dominant syndrome caused by pathogenic variants in one of the DNA MMR genes, namely *MSH2*, *MSH6*, *MLH1*, and *PMS2* [110]. Less frequently, it can be due to deletion of the *EPCAM* gene with subsequent *MSH2* silencing or epigenetic changes, leading to *MLH1* promoter methylation [111,112]. As extensively reviewed in previous studies, loss of MMR gene function leads to the accumulation of mutations and accelerates tumor development. The molecular abnormalities typical of Lynch syndrome are also related to other syndromes, such as Muir–Torre syndrome (which includes sebaceous neoplasms) and Turcot syndrome (which includes brain tumors) [113,114].

Lynch syndrome can be detected either through polymerase chain reaction or NGS-based analysis of microsatellite loci or MMR genes. IHC examination of the proteins encoded by MMR genes may serve as a screening method for germline testing. For a long time, IHC was performed only in patients fulfilling Amsterdam or Bethesda clinical criteria, but nowadays, IHC is indicated for all newly diagnosed CRC cases, regardless of age or history [106]. If loss of MLH1 and/or PMS2 is found, it is necessary to analyze *BRAF* mutations and *MLH1* promoter methylation, as many sporadic cases also lose MMR protein expression without germline mutations. There are many pathogenic variants of the MMR genes associated with LS, but also many VUS that can be found in multiple genomic databases for patient counseling [115]. Likely, pathogenic variants are managed clinically as pathogenic ones. Multigene analysis panels are indicated in cases suspicious of LS, as they are more cost-efficient than isolated gene detection tests.

### 9.5. Follow-Up and Treatment

Periodic follow-up of Lynch syndrome patients with colonoscopy is indicated every 2 years beginning at 20–25 years for *MLH1-* or *MSH2*-associated cases, or at 35 years for *MSH6* and *PMS2* cases. This screening can allow for the prevention and earlier detection of CRC, consequently improving prognosis [116]. The risk of developing both synchronous and metachronous CRC has led some groups to recommend total abdominal colectomy as an alternative for Lynch syndrome patients who have already developed CRC, as it improves quality of life and saves lives [117]. This recommendation is only for carriers of *MSH2* or *MLH1* abnormalities, with segmental colectomy preferred for *MSH6* and *PMS2* mutation carriers. Prophylactic colectomy is not recommended in MMR carriers, as the risk of disease is not 100%. Decisions on surgical therapy should always be based on mutation analysis, not on the results of IHC or *MLH1* promoter methylation [118].

Management of patients with VUS is more complex and still under study. It is necessary to develop individualized management strategies, as VUS can appear in almost 30% of suspected familial cases [119], and they add significant stress on families faced with a situation that neither their doctors nor they can adequately manage at this moment [120].

Evidence suggests that patients with Lynch syndrome can reduce the risk of CRC with the regular use of daily aspirin, adjusting the dose to the risks associated with long-term consumption of this drug [121].

Regarding GC screening, there is no consensus on the use of upper endoscopy. A recent review recommends performing it from the age of 30, and in younger patients if they have gastric symptoms or if they have young family members diagnosed with GC [122].

## 10. Lynch-like Syndromes

Lynch-like syndromes comprise a group of patients who exhibit deficiencies in the DNA MMR system but lack MMR mutations associated with Lynch syndrome. This classification represents a heterogeneous spectrum of cases that evolves continuously with advancements in molecular understanding [100]. Up to 70% of Lynch-like syndrome patients demonstrate loss of IHC expression of one of the MMR proteins, without identifiable germline mutations. Instead, they often exhibit either somatic methylation of the *MLH1* promoter or dual somatic pathogenic changes in MMR genes. Notably, patients with Lynch-like syndromes typically have a lower familial burden of disease and tend to manifest at an older age [100]. However, they require vigilant monitoring for early disease detection and prompt treatment. Thus, in cases where hypermethylation or *BRAF* mutations are absent, it is recommended to conduct colonoscopies biennially from ages 25 to 75 years to mitigate the risk of CRC [121].

Several syndromes share similarities with Lynch syndrome but possess distinct genetic characteristics, including constitutional mismatch repair deficiency syndrome and familial CRC type x syndrome.

### 10.1. Constitutional Mismatch Repair Deficiency Syndrome

This autosomal recessive disorder is marked by homozygous or compound heterozygous germline pathogenic variants of MMR genes [123]. Constitutional mismatch repair deficiency syndrome ranks among the most aggressive cancer predisposition syndromes, with patients developing CRC and other malignancies, including hematological and solid tumors, at a remarkably young age. Screening protocols typically start at age 8, and surveillance includes brain magnetic resonance imaging due to the heightened susceptibility to brain tumors. Despite challenges in genetic testing interpretation, an international consortium of experts has recently established clinical, genetic, and ancillary criteria to facilitate diagnosis, as surveillance and early intervention are critical for prognosis [124].

### 10.2. Familial Colorectal Cancer Type X Syndrome

This autosomal dominant hereditary syndrome meets the clinical criteria for Lynch syndrome but lacks germline mutations in MMR genes or MSI [125]. The genetic basis of familial CRC type x syndrome remains poorly elucidated, with only the *RSP40* gene definitively linked to the disease, though numerous potential candidates are under investigation [126,127]. While ongoing studies aim to uncover more cases of familial CRC type x syndrome, evidence suggests that the progression rate from adenomas to carcinomas is slower, allowing for surveillance every 3–5 years commencing at the age of the youngest affected relative [125].

## 11. Classic and Attenuated Familial Adenomatous Polyposis

This group of hereditary polyposis CRC syndromes with adenomatous polyps is characterized by the development of colorectal adenomas that progress to CRC. While the molecular basis of familial adenomatous polyposis has been known for some time, it accounts for only a small percentage of cases. The widespread use of NGS has led to an explosion of knowledge about other pathogenic variants that cause similar clinical findings, resulting in what is termed *APC*-negative adenomatous polyposis [128].

The first reported case of familial adenomatous polyposis dates back to 1881 by Sklifasowski, with Cripps proposing hereditary risk for the development of multiple polyps in 1882. Over subsequent years, numerous authors contributed cases and families to this emerging entity, establishing a high risk of malignant transformation of these polyps and their association with similar polyps throughout the GI tract. In 1939, Dukes published data from 10 families followed in the first Polyposis Registry at St. Mark’s Hospital [129]. The genetic basis of the disease was elucidated in 1986 when Herrera et al. described an abnormality in chromosome 5q, and in 1987, two independent groups identified the *APC* gene mapped to 5q21-22 as the molecular abnormality underlying familial adenomatous polyposis [130].

### 11.1. Clinical Presentation

This syndrome is characterized by the presence of a large number of polyps in the large bowel, with rectal bleeding being the most common symptom leading to diagnosis. However, some cases are identified through genetic screening. Polyps typically develop in childhood, and at the time of diagnosis, 60% of patients have more than 50 polyps along the colon. Other symptoms associated with *APC* mutations include hypertrophy of the retinal pigment epithelium; desmoid tumors (Gardner syndrome); osteomas; epidermal cysts; supernumerary teeth; and GI carcinomas, including CRC, small bowel cancer, and pancreatic cancer [131]. The phenotype appears to be influenced by the location of *APC* mutations, leading to two distinct clinical scenarios: classical familial adenomatous polyposis (including profuse familial adenomatous polyposis with over 1000 adenomas along the colonic mucosa and sparse familial adenomatous polyposis with 100–1000 polyps) and attenuated familial adenomatous polyposis with 10–99 adenomas, predominating in the right colon [132].

### 11.2. Risk of Colorectal Cancer

Although familial adenomatous polyposis is the most common cause of polyposis, it explains less than 1% of all CRC cases. Early reports showed that malignancy develops in classical familial adenomatous polyposis before the ages of 40–50 in almost 100% of cases, but more recent data suggest lower rates of cancer due to the adoption of preventive measures [133,134]. This risk may also vary depending on the location of *APC* mutations [135].

### 11.3. Molecular Basis

The *APC* gene, located on chromosome 5q, acts as a tumor suppressor gene, and its loss of function leads to the activation of the Wnt pathway, loss of cell adhesion, defects in cell cycle control, deregulation of base excision repair (BER), and impairment of chromosomal segregation [136]. The BER mechanism will be discussed in more detail in the section on *MUTYH*-associated polyposis. Mutations involving codons 1250–1464 of the *APC* gene are associated with classical familial adenomatous polyposis. Attenuated forms result from pathogenic or likely pathogenic variants in the 5′ or 3′ ends of the gene and selective splicing of exon 9 [137]. The location of the *APC* mutation also influences extracolonic manifestations of the disease. However, *APC* pathogenic variants are found in only 60% of clinically suspected cases (termed *APC*-negative polyposis), some of which are explained by other molecular abnormalities [132]. Multipanel testing is recommended, but single-site tests can be used if a pathogenic variant has already been confirmed in a given family.

### 11.4. Follow-Up and Treatment

Traditionally, patients with familial adenomatous polyposis were recommended proctocolectomy in their 20s to prevent CRC. However, this approach has evolved with the understanding of genotype–phenotype correlations, allowing for postponing surgery or using less invasive and aggressive surgical options. Nevertheless, surgery remains the best therapeutic option for these patients [121]. From the age of 12, familial adenomatous polyposis patients should undergo colonoscopies every 1–3 years according to the phenotype. After colectomy, pouchoscopy is recommended every 1–3 years, depending on the phenotype [132].

## 12. *MUTYH*-Associated Polyposis

*MUTYH*-associated polyposis is an autosomal recessive syndrome characterized by biallelic pathogenic variants in the *MUTYH* gene. Patients with *MUTYH*-associated polyposis typically develop numerous colorectal polyps, although rarely exceeding 100. Unlike some other syndromes, extraintestinal manifestations are infrequent in this entity, and primarily involve the proximal small bowel [138]. The risk of CRC can be as high as 90% in biallelic carriers, while monoallelic carriers exhibit only a slightly increased risk compared to the general population and often lack a polyposis phenotype. Therefore, biallelic carriers require regular annual colonoscopies from a young age (18–20 years). Interestingly, previous reports have found CRC even in patients with a low number of polyps [136]. Recent findings have suggested an increased risk of other malignant tumors, such as breast, pancreas, ovary, and bladder cancers, in monoallelic *MUTYH* mutation carriers, prompting the proposal to rename the condition *MUTYH*-associated tumor syndrome [139].

Patients under 60 years with 10 or more adenomas, and those over 60 with 20 or more adenomas or 10 or more adenomas with a family history of adenomas or CRC but without pathogenic variants in *APC* or *MUTYH* are categorized as having “multiple colorectal adenomas” [140]. In these cases, guidelines recommend 1–2 colonoscopies per year from the time of diagnosis until 75 years of age to reduce CRC risk, along with germline panel testing to exclude potential genetic abnormalities.

### Molecular Basis

The *MUTYH* gene, located on chromosome 1, plays a crucial role in DNA repair, specifically in the BER mechanism. With the advent of NGS analysis, our understanding of the BER process, in which DNA glycosylases like MUTYH initiate DNA repair upon detection of base abnormalities, has significantly improved. This system repairs various forms of oxidative, deamination, alkylation, and abasic single-base damage, which may have minimal effects on the helix but are associated with significant processes such as carcinogenesis, inflammation, aging, or the development of neurodegenerative disorders [141].

The BER mechanism aids in repairing damaged DNA through two primary pathways: short-patch and long-patch. In the short-patch BER pathway, a single nucleotide is replaced in the repair process, while the long-patch BER pathway involves the replacement of at least two nucleotides. As previously mentioned, initiation of the BER pathway is facilitated by DNA glycosylases, responsible for recognizing and removing damaged bases. Subsequently, the completion of the BER pathway requires the involvement of at least three additional enzymes, which carry out tasks such as strand incision, gap-filling, and ligation [142].

## 13. *NTHL1*-Associated Tumor Syndrome

The *NTHL1* (Nth-like DNA glycosylase 1) gene encodes another DNA glycosylase that, similarly to MUTYH, plays a role in the BER process. This syndrome follows a recessive mode of inheritance [143]. Biallelic mutations in *NTHL1* predispose individuals to polyposis, CRC, and numerous other tumor types, earning it the name *NTHL1*-associated tumor syndrome [144]. An initial description of a polyposis syndrome in 7 cases with adenomatous polyps identified a germline mutation in p.Q90* [145]. Subsequent studies revealed either homozygous mutations in p.Q90* or a heterozygous combination of p.Q90*/p.287* [146].

A recent review showed that the risk of CRC in patients with this syndrome is high, occurring in 49% of cases, with a mean age at diagnosis of 55 years [147]. Among heterozygous carriers, 18% were diagnosed with CRC. Tumors most frequently associated with this syndrome, in addition to CRC, include those of the breast and endometrium, suggesting that along with colonoscopic surveillance, periodic breast and gynecological examinations would be advisable in these patients [148].

### Molecular Basis

The *NTHL1* gene is located on the short arm of chromosome 16 and encodes the protein of the same name [149]. In contrast to MUTYH, NTHL1 is bifunctional, as it can perform both glycosylase and lyase functions, whereas MUTYH only exhibits glycosylase activity [143]. During the BER process, bifunctional glycosylases remove the abnormal nucleotide base, cleave the DNA strand, and create a single-stranded gap harboring modified 3′ ends, which is remodeled by other enzymes. Thus, the dysfunctional *NTHL1* protein leads to genomic instability and increases the risk of cell transformation.

Interestingly, research is ongoing to delineate the pathogenic role of single nucleotide polymorphisms and other genomic alterations of the *NTHL1* gene that could influence cancer development and aging, likely through telomere length control [143].

## 14. Polymerase Proofreading-Associated Polyposis

Polymerase proofreading-associated polyposis is an autosomal dominant syndrome associated with a high risk of CRC and other malignancies, including brain, duodenal, and breast carcinomas [150]. It is caused by germline pathogenic variants of the *POLD1* gene (chromosome 19) and *POLE* gene (chromosome 12). These genes encode the catalytic subunits of polymerases delta and epsilon, respectively, which are involved in DNA proofreading to ensure replication fidelity. Pathogenic variants in these genes lead to the accumulation of thousands of mutations, resulting in an ultramutated phenotype [151]. Patients develop GI polyps and have an increased risk of malignant transformation, with CRC risk approaching 90% for *POLD1* variant carriers and nearly 30% for *POLE* variant carriers. Despite the better prognosis of ultramutated tumors and their favorable response to immune checkpoint inhibition, affected individuals should undergo stringent surveillance from a young age [137].

## 15. Axis Inhibition Protein 2 *(AXIN2)*-Associated Polyposis

*AXIN2*-associated polyposis is an autosomal dominant syndrome characterized by colorectal polyps and oligodontia, defined as a congenital absence of at least seven teeth. However, a recent French series involving 11 unrelated individuals with *AXIN2* variants revealed that oligodontia was present in only 60% of cases. Nonetheless, this report confirmed that loss-of-function variants of this gene increase the risk of CRC and other GI neoplasms, with 40% of patients developing malignant disease and 91% exhibiting colorectal polyposis [152]. The prevalence of these variants in the report was notably low, at 0.24% [153]. Despite its rarity, *AXIN2* presents an intriguing potential target for boosting immunotherapy [154].

**Figure 7:**
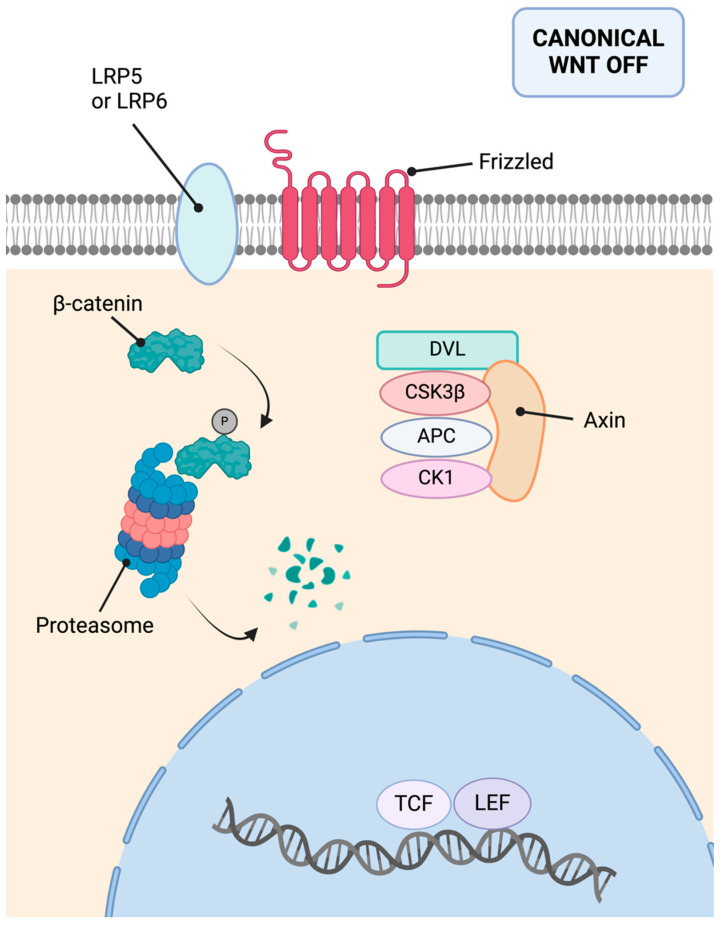
Inactive canonical Wnt pathway. The absence of Wnt ligands leads to the phosphorylation of β-catenin by the destruction complex, comprising Axin, APC, and several kinases. This phosphorylated β-catenin is subsequently degraded by the proteasome. Additionally, without nuclear β-catenin, a repressive complex (TCF-TLE) inhibits gene transcription. Image created with Biorender.com (https://www.biorender.com/, accessed on 1 May 2024) under an individual license (Dr. Díaz del Arco).

Regarding its molecular basis, the *AXIN2* gene is a tumor suppressor gene located on chromosome 17q24. It encodes the Axin-2 protein, also known as conductin [155]. This protein is crucial as a negative regulator of the Wnt pathway. It acts as a scaffold for the β-catenin destruction complex, facilitating β-catenin degradation [153]. Consequently, it inhibits the pro-proliferative function of the Wnt pathway. Figure 7 summarizes the inactive canonical Wnt pathway.

## 16. Dual Oxidase 2 *(DUOX2)*-Associated Polyposis

*DUOX2* has traditionally been linked to thyroid function, as it has been shown to contribute to congenital hypothyroidism due to thyroid dysgenesis [156]. However, it also appears to play a role in maintaining normal GI microbiota and has recently been suggested as a candidate gene for *APC*-negative familial polyposis [157]. In the GI tract, DUOX2 alterations are rare but have been associated with the occurrence of CRC and increased tumor aggressiveness [158]. These alterations seem to cause endoplasmic reticulum retention with an unfolded protein response, which promotes adenomatous changes and carcinogenesis. Both missense and nonsense mutations of the *DUOX* gene lead to the overexpression of a truncated pathogenic protein [159]. The clinical relevance and management strategies for these variants are still not well defined.

## 17. Other Candidate Genes in Adenomatous Polyposis Syndromes

NGS studies of different cohorts of *APC*-negative polyposis syndrome patients are leading to the identification of many gene variants associated with these conditions [160,161]. Studies are ongoing to evaluate the potential pathogenic role of genes such as *MLH3*, *MSH3*, *GALNT12*, *ATM*, and *SDHA*, among others [162]. In the near future, expanding knowledge about these and other candidate genes will enable the early management of more patients.

## 18. Hereditary Polyposis Colorectal Carcinoma with Serrated Polyps

Serrated polyps, which include hyperplastic polyps (usually left-sided), sessile serrated polyps (usually right-sided), and serrated adenomas, are confirmed through histopathological analysis. Though once considered rare, serrated polyposis syndrome is now recognized as the most prevalent polyposis syndrome leading to CRC. It is characterized by the development of multiple serrated polyps throughout the colon and rectum. The inheritance pattern of serrated polyposis syndrome remains unclear, yet first-degree relatives of patients seem to face a significantly increased risk of CRC and pancreatic cancer [163].

### 18.1. Clinical Presentation

The World Health Organization defines serrated polyposis syndrome by two possible clinical criteria. A diagnosis can be established if a patient has at least five serrated polyps, each measuring 5mm or larger, located proximal to the rectum with at least two of these polyps being 10mm or larger. Alternatively, a diagnosis may be made when a patient presents with more than 20 serrated polyps anywhere along the bowel, with at least five of these being proximal to the rectum [164]. The average age at diagnosis is around 50 years, and less than half of the patients report a familial history of the disease, highlighting the ongoing debate regarding its hereditary nature.

The incidence of CRC in patients with serrated polyposis syndrome varies but is noted to be over 20% according to various studies. The highest risk occurs at the time of diagnosis (10–20%) and tends to decrease during follow-up (2–4%) [165].

### 18.2. Molecular Basis

Serrated polyposis syndrome appears to be a heterogeneous condition, characterized by an activating mutation in the *BRAF* gene, which may or may not be associated with MSI [164]. Although the progression risk is well documented, less than 5% of serrated polyposis syndrome cases have been linked to germline pathogenic variants. A review by Murphy et al. indicated significant overlap with other polyposis syndromes; some patients with pathogenic variants in genes like *MUTYH*, *PTEN*, *SMAD4*, *BMPR1A*, *POLE*, and *GREM1* presented features of both serrated polyposis syndrome and other syndromes [166]. Therefore, there is controversy regarding its molecular basis, and some authors emphasize that many cases have no family history of polyposis and a strong association with environmental factors has also been observed [167].

### 18.3. Follow-Up and Treatment

Genetic testing is recommended for younger patients, those with multiple affected relatives, or those who have dysplasia in any polyps. Management primarily involves regular, high-quality colonoscopies. The recommended colonoscopy interval is every 1–2 years, with removal of all polyps if feasible, or at least those 5 mm or larger or those suspected of having dysplastic changes [168]. In cases where endoscopic control is impractical due to numerous lesions, radical surgery (colectomy) may be necessary. First-degree relatives should undergo colonoscopy every 5 years, starting from the age of 45 years [169].

### 18.4. RNF43-Associated Polyposis

Recent studies underscore the tumor suppressor role of the *RNF43* gene [170,171,172]. RNF43 is a U3 ubiquitin ligase with multiple anti-carcinogenic functions: it is responsible for ubiquitinating and stabilizing p53, regulates the cell cycle, and inhibits the Wnt and Hippo pathways [173]. Germline variants in *RNF43*, considered likely pathogenic, have been identified in families characterized by *BRAF*-mutated CRC linked to the serrated pathway and exhibit an autosomal dominant pattern of inheritance [170]. *RNF43* is emerging as a promising candidate gene for explaining familial predisposition to serrated polyposis syndrome and is also being explored as a potential marker for targeted drug development [174]. However, the precise role of *RNF43* in CRC tumorigenesis remains to be fully determined.

## 19. Juvenile Polyposis Syndrome

Juvenile polyposis syndrome is an autosomal dominant cancer syndrome characterized primarily by the development of numerous juvenile polyps in the GI tract, particularly in the colon and rectum, where they are present in 98% of cases [175]. Despite its name, “juvenile” refers to the histological appearance of the polyps rather than the age of onset. The average age at diagnosis is 18 years.

The number of polyps in juvenile polyposis syndrome patients can range from a few to over 100 [176]. The syndrome is suspected when a patient has five or more juvenile polyps in the colon (especially in the proximal section), a juvenile polyp in the proximal GI tract, or any number of juvenile polyps along with a family history of juvenile polyposis syndrome. Extraintestinal manifestations include pigmented nevi, telangiectasias on the skin, and various skeletal abnormalities [177]. Patients with juvenile polyposis syndrome face a high lifetime risk of developing colorectal cancer, which can reach up to 70% [175].

More than one-third of juvenile polyposis syndrome cases arise from de novo mutations and frequently mutated genes include Bone Morphogenetic Protein Receptor 1A (*BMPR1A*) or Suppressor Mothers Against Decapentaplegic Homolog 4 (*SMAD4* or *DPC4*) [178]. *BMPR1A*, a serine-threonine kinase type I receptor, regulates the Transforming Growth Factor β (TGF-β) pathway, crucial for cell growth inhibition and apoptosis [179]. Juvenile polyposis syndrome-associated abnormalities typically include large deletions and point mutations, occasionally involving the gene promoter, and affected patients tend to show a large bowel-predominant phenotype. *SMAD4*, identified as a tumor suppressor gene in 1996, is an essential mediator of TGF-β pathway [180]. Mutations in *SMAD4* are linked to both colonic and gastric polyps and to hereditary hemorrhagic telangiectasia, with high penetrance, leading to the development of colonic polyps in 97% of affected individuals before the age of 50 and increasing risks for gastric and pancreatic cancers.

For patients with a confirmed diagnosis of juvenile polyposis syndrome who lack the typical pathogenic variants, extensive genomic evaluations may uncover other significant genetic mutations [181,182]. Surveillance recommendations include annual colonoscopy starting at 12 years old, though the starting age and frequency may be adjusted based on polyp burden and symptoms [183]. All polyps larger than 5 mm should undergo removal and histological examination. Colectomy is contemplated only for patients with numerous polyps that are unmanageable through endoscopic approaches.

### Juvenile Polyposis of Infancy

Juvenile polyposis of infancy, also known as chromosome 10q22.2-q23.2 deletion syndrome, represents a severe subtype of juvenile polyposis syndrome [184]. Linked to microdeletions in the *BMPR1A* gene, juvenile polyposis of infancy is characterized by early onset before age 2, severe enteropathy, and high mortality [185]. The 10q22.2-q23.2 deletion often involves the *PTEN* gene, which is also associated with Cowden disease. Advances in genomic technology have started to uncover small copy number variations that contribute to the molecular basis of juvenile polyposis of infancy.

## 20. Peutz–Jeghers Syndrome

Peutz–Jeghers syndrome is an autosomal dominant disorder characterized by hamartomatous GI polyposis, mucocutaneous pigmentation, and a predisposition to cancer, caused by germline pathogenic variants in the *STK11* gene [186]. Hamartomatous polyps can occur throughout the GI tract but are most commonly found in the small intestine, particularly the jejunum [187]. The most frequent types of cancer in Peutz–Jeghers syndrome patients include breast and GI cancers, specially CRC. Peutz–Jeghers syndrome is also associated with tumors of the liver, lungs, ovaries, uterus, and testicles [188]. The syndrome is rare, with an estimated incidence of 1 in 50,000 to 200,000 births [189].

The diagnostic criteria for Peutz–Jeghers syndrome are (1) two or more histologically confirmed Peutz–Jeghers-type polyps, (2) any number of Peutz–Jeghers-type polyps in an individual with a first-degree relative with Peutz–Jeghers syndrome, (3) characteristic mucocutaneous pigmentation in a person with a family history of Peutz–Jeghers syndrome, or (4) any number of Peutz–Jeghers-type polyps and characteristic mucocutaneous pigmentation [53].

### 20.1. Clinical Presentation

The average age at diagnosis is between 18 and 23 years [190,191]. Characteristic pigmentation appears in 95% of patients as small dark blue, black, or brown mucocutaneous macules, visible during childhood from around 5 years of age and potentially fading during puberty, although perioral lesions often persist [189]. These macules typically appear around the eyes, nostrils, and perioral area, as well as on the palms, soles, fingers, and perianal area [192]. They are not usually associated with the development of malignant melanotic tumors.

Peutz–Jeghers intestinal polyps are hamartomatous and characterized by an arborizing pattern of smooth muscle cells between mucosal epithelial compartments. Gastric polyps may exhibit less specific histological features, though classic cases have been documented [193,194]. GI polyps can lead to chronic bleeding, anemia, and recurrent bowel obstruction and intussusception [195].

### 20.2. Colorectal Cancer Risk

Patients with Peutz–Jeghers syndrome have a significantly increased risk of cancer (15–18 times higher than the general population), with a mean onset age of 41 years and a cumulative probability of 55% by age 60 [189,196]. The lifetime risk for CRC is estimated at 39% [197]. Regarding GC, data on its occurrence in Peutz–Jeghers syndrome are limited. A previous study showed a high risk of GC, with a cumulative probability of 29% between the ages of 15 and 64 years [198].

### 20.3. Molecular Basis

Peutz–Jeghers syndrome is caused by germline mutations in the *STK11* gene, combined with an acquired second-hit defect [199]. This gene is located on chromosome 19 (19p13.3) and consists of nine coding exons (1302 base pairs) and one non-coding exon [200]. It encodes the LKB1 protein, which has 433 amino acids and a molecular weight of 50 kDa [201]. The protein comprises an amine-terminal domain, a kinase domain, and a carboxy-terminal regulatory domain [202].

The *STK11* gene acts as a tumor suppressor, and its defect can make cells particularly vulnerable to energetic stress mediated by the AMP-activated protein kinase [203]. This kinase also regulates cell cycle, cell differentiation, apoptosis, and DNA damage repair [204]. Additionally, LKB1 inhibits the mTOR pathway [205]. This pathway is crucial in cancer development, because it promotes tumor growth, cell proliferation, metabolism, and immune tolerance, while inhibiting autophagy and apoptosis [206]. Activation of the mTOR pathway has pro-oncogenic effects, and *STK11* dysfunction can trigger these effects. Indeed, previous studies have shown that loss-of-function mutations in *STK11* reduce response to immunotherapy in patients with non-small cell lung cancer [207]. In GI cancer, the association of *STK11* mutations with treatment resistance is less clear [208].

Apart from these roles, LKB1 is involved in chromosomal segregation, inhibits the TGF-β and Wnt signaling pathways, and regulates p53 activity [209]. By inhibiting these pathways and mediating p53 activity, it further fulfills its tumor suppressor functions [210,211]. The roles of the Wnt pathway and p53 protein have been discussed in more detail in the sections on hereditary diffuse GC and Li–Fraumeni syndrome (see above). The TGF-β pathway has protumorigenic effects, promoting EMT and enhancing cellular plasticity and survival [212]. TGF-β has a dual effect, inducing apoptosis in early stages and promoting tumor growth in later stages, increasing aggressiveness, invasiveness, and metastasis [213]. TGF-β acts not only at the tumor cell level but also within the tumor microenvironment, including immune and stromal components [214].

### 20.4. Follow-Up and Treatment

According to the European Society of Gastrointestinal Endoscopy, the first endoscopic examination (oesophagogastroduodenoscopy and colonoscopy) is recommended at 8 years of age, with follow-up intervals depending on the presence of symptoms and identification of polyps [215]. Polyps larger than 15–20 mm, or smaller polyps causing symptoms, should be resected.

## 21. Cowden Syndrome

Cowden syndrome was first described in 1963 as an autosomal dominant disorder. It is characterized by multiple hamartomas affecting various organs and an increased risk of several tumors, including breast, thyroid, or uterine cancer [216]. The diagnostic criteria for Cowden syndrome were established in 1996 [217]. Most patients begin to exhibit cutaneous symptoms during adolescence, and approximately 50–70% of cases develop thyroid abnormalities [218]. Additionally, up to 93% of patients undergoing colonoscopy reveal polyps, predominantly hamartomatous, although hyperplastic polyps, ganglioneuromas, and leiomyomas are also common [219]. It is typical for these patients to present with several different types of polyps synchronously. Cowden syndrome may also be associated with dysplastic gangliocytoma (Lhermitte–Duclos disease) and can overlap with Bannayan–Riley–Ruvalcaba syndrome [220,221]. The clinical diagnosis can be challenging, with genetic studies playing a crucial role in the diagnostic process [185].

### 21.1. Colorectal Cancer Risk

Traditionally, it was believed that patients with Cowden syndrome were prone to developing thyroid and endometrial cancers but not at an increased risk for CRC. However, recent studies suggest otherwise, estimating that the risk of CRC could be as high as 13–15%, with all cases occurring before the age of 50 [222,223].

### 21.2. Molecular Basis

Cowden syndrome is linked to mutations in the phosphatase and tensin homolog (*PTEN*) gene located on chromosome 10, which encodes a protein tyrosine phosphatase. *PTEN* is considered a tumor suppressor gene, as it inhibits the mTOR pathway and plays important roles in cell growth, proliferation, survival, DNA repair, and cell motility [224]. Various genetic alterations, including nonsense and missense mutations, deletions–insertions, and splice site mutations, have been described in up to 81% of Cowden syndrome patients, affecting the entire length of the gene, predominantly spanning exon 1 [225]. Notably, about 47% of Cowden syndrome cases arise from de novo mutations [226].

### 21.3. Follow-Up and Treatment

For Cowden syndrome patients, colonoscopy is recommended to begin at age 35. If polyps are detected, subsequent colonoscopies should be scheduled annually; if none are found, every five years is sufficient. If a family member develops CRC, surveillance should start 5–10 years prior to the age at which the relative was diagnosed. Colectomy is indicated only in select cases, based on clinical findings [226].

### 21.4. PTEN Hamartoma Tumor Syndrome

*PTEN* hamartoma tumor syndrome encompasses conditions related to *PTEN* germline mutations, such as Proteus syndrome and Bannayan–Riley–Ruvalcaba syndrome, and is often discussed interchangeably with Cowden syndrome [227]. However, no clear genotype–phenotype correlation has been established. The revised criteria for diagnosing this syndrome include major and minor criteria [228]. Among the major criteria are the detection of breast, endometrial, or thyroid cancers; GI hamartomas; and other lesions such as macrocephaly, acral keratoses, mucocutaneous neuromas, and oral papillomas.

## 22. Hereditary Polyposis with Mixed Polyps Syndrome

Hereditary polyposis with mixed polyps syndrome is characterized by the development of polyps with histologically mixed features. This can involve polyps showing histologically different areas or the presence of multiple polyps of different types in the same patient, though a strict quantitative definition is still lacking. At least two distinct forms of the disease have been described [229].

### 22.1. GREM1-Associated Mixed Polyposis

Gremlin 1 (*GREM1*) encodes a member of the bone morphogenetic protein antagonist family. It exerts its antagonistic effect by directly binding to bone morphogenetic proteins, influencing body patterning and tissue differentiation. Its role in cancer is not clear, as it exerts both anti-tumorigenic and pro-tumorigenic effects, favoring stemness, activation of cancer-associated fibroblasts, angiogenesis, and cell proliferation [230].

Cenani–Lenz syndrome, characterized by oligosyndactyly, metacarpal synostosis, and phalangeal disorganization with additional facial features, is mostly associated with *LRP4* mutations [231]. However, some cases have been linked to truncating mutations in the *APC* gene, mutations in formin 1 (*FMN1*), or rearrangements of the *GREM1-FMN1* loci. It is worth noting that this last molecular alteration has been associated with an increased risk of CRC through mechanisms yet to be fully elucidated. Additionally, this occurs more frequently in diabetic and obese patients, highlighting the interplay between environmental and genetic factors in cancer development [232].

### 22.2. BMPR1A-Associated Mixed Polyposis

Bone morphogenetic protein receptor type 1A (*BMPR1A*) has been linked to juvenile polyposis syndrome and, more recently, to a syndrome with overlapping features known as hereditary mixed polyposis syndrome 2. Patients with hereditary mixed polyposis syndrome 2 exhibit germline mutations, including frame-shift, nonsense, or missense mutations in the *BMPR1A* gene. They display a variable phenotype characterized by polyps with mixed histological features and, in some cases, the development of CRC without prior polyps [233].
